# Effects of a cardioprotective nutritional program on apolipoproteins and lipids in secondary cardiovascular disease prevention

**DOI:** 10.20945/2359-4292-2024-0373

**Published:** 2025-03-11

**Authors:** Aline Marcadenti, Josefina Bressan, Annie Seixas Bello Moreira, Rachel Helena V. Machado, Renato Hideo N. Santos, Cristiane Kovacs Amaral, Marcelo Macedo Rogero, Vinícius Cooper Capetini, Angela C. Bersch-Ferreira

**Affiliations:** 1 Instituto de Pesquisa Hcor, São Paulo, SP, Brasil; 2 Programa de Pós-graduação em Ciências da Saúde (Cardiologia), Instituto de Cardiologia/Fundação Universitária de Cardiologia do Rio Grande do Sul, Porto Alegre, RS, Brasil; 3 Programa de Pós-graduação em Epiemiologia, Faculdade de Saúde Pública, Universidade de São Paulo, São Paulo, SP, Brasil; 4 Departamento de Nutrição, Faculdade de Saúde Pública, Universidade de São Paulo, São Paulo, SP, Brasil; 5 Departamento de Nutrição e Saúde, Universidade Federal de Viçosa, Viçosa, MG, Brasil; 6 Departamento de Medicina Translacional, Faculdade de Ciências Médicas, Universidade Estadual de Campinas, Campinas, SP, Brasil; 7 Departamento de Nutrição Clínica, Universidade do Estado do Rio de Janeiro, Rio de Janeiro, RJ, Brasil; 8 Ambulatório de Nutrição Clínica do Instituto Dante Pazzanese de Cardiologia, São Paulo, SP, Brasil; 9 Gabinete PROADI-SUS do Hospital Beneficência Portuguesa, São Paulo, SP, Brasil

**Keywords:** Cardiovascular diseases, secondary prevention, apolipoproteins, diet, healthy

## Abstract

**Objective:**

This study aimed to evaluate the impact of the Brazilian Cardioprotective Nutrition Program (BALANCE Program) on the plasma levels of various apolipoproteins (A-I, A-II, B, C-II, C-III, and E) and lipid biomarkers over a three-year follow-up period in individuals undergoing secondary cardiovascular prevention.

**Subjects and methods:**

This exploratory analysis included 276 patients aged 45 years or older with a history of cardiovascular disease within the preceding decade. Participants were randomly assigned to one of two groups and monitored over three years: the BALANCE Program group (intervention group; n = 123) and the control (conventional nutritional advice; n = 153). Assessments of clinical and lifestyle data, anthropometry, food intake, plasma apolipoproteins, and lipid profiles were conducted at baseline and at the 3-year follow-up. Intervention adherence was measured utilizing the BALANCE dietary index.

**Results:**

By the end of the follow-up period, adherence was significantly higher in the intervention group (mean difference BALANCE-control [95% CI]: 2.09 points [-0.19; 4.37]), mainly due to increased consumption of fruits, vegetables, legumes, and low-fat dairy products. There were no significant differences in plasma apolipoprotein levels between the groups throughout the study. Nevertheless, significant reductions were observed in the total cholesterol and non-HDL cholesterol levels in the BALANCE group compared to the control group (mean difference intervention-control [95% CI]: -9.95 mg/dL [-18.5; -1.39] and -8.86 mg/dL [-17.53; -0.2], respectively).

**Conclusion:**

Following three years of intervention, despite higher adherence to the BALANCE Program, there were no significant changes in plasma apolipoprotein concentrations or overall lipid biomarkers.

## INTRODUCTION

Cardiovascular disease (CVD) is the leading cause of mortality worldwide (^1^). Modifiable risk factors, including body weight, physical activity, smoking, and diet, are primary targets for its treatment and prevention (^2^). In this regard, the cardioprotective effects of diets such as the Mediterranean and Dietary Approaches to Stop Hypertension (DASH) diets in reducing cardiovascular risk are well-established (^3^,^4^). These diets are considered key references for dietary guidelines aimed at managing and preventing CVD (^5^).

Research has shown that apolipoproteins (Apo) and ratios such as ApoB/ApoA-I may predict CVD risk more accurately than traditional lipid measures (^6^). Randomized clinical trials have demonstrated that dietary patterns such as the DASH and Mediterranean diets can influence Apos in primary cardiovascular prevention (^7^-^9^). The beneficial effects of these diets on blood lipid profiles can be attributed to the increased intake of unsaturated fatty acids and bioactive compounds found in fruits, vegetables, olive oil, and nuts - typical components of these dietary patterns (^10^,^11^).

Despite their cardiovascular benefits, adherence to both the Mediterranean and DASH diets is often lower due to cultural, social, and economic factors among the general population (^12^,^13^). To address this, the development of dietary approaches with affordable ingredients, while adhering to nutritional guidelines, could enhance compliance. The Brazilian Cardioprotective Diet Program (BALANCE Program) was developed to provide an accessible nutrition education tool for the population, incorporating recommendations for managing CVD to improve patient understanding of dietary prescriptions and adherence (^14^-^16^).

Although the BALANCE Program has been evaluated for its impact on various CVD-related biomarkers (^15^,^17^) and is recommended for controlling multiple cardiovascular risk factors (^16^), its effects on Apos have not yet been assessed. Moreover, most randomized trials evaluating the effects of dietary patterns on Apos have focused on populations in primary cardiovascular prevention (^7^-^9^,^18^,^19^). Given the markedly different risk profiles between these populations, understanding the behavior of Apos in secondary prevention is essential for tailoring appropriate care and treatment strategies for this group. Patients undergoing secondary prevention have not only a higher cardiovascular risk but are also often managing multiple medications, complicating the interpretation of the isolated effects of interventions such as dietary behavior changes in this multi-drug context. Nonetheless, understanding these effects is crucial for assessing the potential adjuvant and protective benefits of such interventions.

Given this context, this study sought to evaluate the impact of the BALANCE Program on the concentrations of Apos A-I, A-II, B, C-II, C-III, and E in subjects undergoing secondary prevention for CVD, after three years of intervention, as our primary outcome. Secondary outcomes included evaluating the BALANCE Program’s effects on other lipid features.

## SUBJECTS AND METHODS

Participants enrolled in the BALANCE Program, a parallel-group, multicenter, randomized, controlled clinical trial with a 1:1 allocation ratio. This trial aimed to evaluate the effects of the BALANCE Program on the secondary prevention of CVD (^14^,^15^). From March 5, 2013, to April 7, 2015, a total of 2,534 participants were randomly assigned to the BALANCE Program, with follow-up concluding on October 31, 2017. All participants provided written informed consent prior to finalization as participants (^15^). The study protocol received approval from the Hcor Ethics Committee (CAAE no. 03218512.0.1001.0060) and the Local Ethics Committee, conducted in accordance with the Helsinki Declaration principles. The BALANCE Program is registered with ClinicalTrials.gov (identifier no. NCT0162039) and conformed to Consolidated Standards of Reporting Trials (CONSORT) guidelines (^20^).

Eligibility criteria were delineated in the study protocol (^14^). In summary, eligible participants were individuals aged 45 years or older, in the secondary prevention phase of CVD, having experienced coronary disease, stroke, or peripheral vascular disease within the preceding ten years. Participants were randomly allocated to either the BALANCE Program group or the Control group. Randomization was stratified by study site and performed in blocks, with allocation concealment secured through a 24-hour central web-based automated system. Blinding was maintained for statisticians, data managers, and laboratory staff only.

The initial sample for this exploratory analysis comprised volunteers from the Dante Pazzanese Institute of Cardiology and the State University of Rio de Janeiro, enrolling 682 participants in the BALANCE study. Of these, 142 volunteers were excluded due to missing dietary data, and 264 were excluded for lacking baseline and 3-year plasma samples.

Participants in the Control group received general dietary advice from dietitians, focusing on a low-fat, low-energy, low-sodium, and low-cholesterol diet. The dietary recommendations were qualitative, not specifying targets for energy and macronutrient intake (^14^,^15^).

The experimental group (i.e., the BALANCE Program group) received an intervention detailed in the study protocol (^14^). The BALANCE Program diet was aligned with the nutritional recommendations from the Brazilian Cardiovascular Guidelines, incorporating elements from the Mediterranean and DASH diets (^21^,^22^). The program emphasized a sustainable dietary prescription adhering to the Brazilian Cardiovascular Guidelines, nutritional education through engaging and interactive strategies promoting affordable foods, and intensive follow-up through individual and group sessions, as well as phone contacts.

To implement the nutritional guidelines and menu suggestions, foods were categorized by nutrient density. Foods meeting criteria for energy density, saturated fatty acids, dietary cholesterol, and sodium density were classified into color-coded groups: “green” (fruits, vegetables, legumes, low-fat dairy), “yellow” (grains, rice, bread, vegetable oils, honey), “blue” (meat, eggs, fish, poultry, cakes, butter), and “red” (trans fats, artificial sweeteners, preservatives). The dietary guide, referring to the colors of the Brazilian flag, advocated predominant intake from the green group, limited intake from the yellow group, and avoidance of the blue and red groups. Menus ranging from 1,400 to 2,400 calories (in 200-kcal intervals) were devised to enhance adherence, supplemented by a regional Brazilian recipe cookbook for educational purposes. The definition of food groups and menu composition are detailed in the study protocol (^14^).

Trained interviewers collected demographic and clinical characteristics, smoking status, physical activity, anthropometric measures, comorbidities, and medication use using a structured questionnaire. Data were recorded in an electronic case report form.

Plasma Apo levels, the primary outcomes, were measured in mg/dL using a multiplex immunoassay (Milliplex, Merck Millipore, USA) as per the manufacturer’s instructions. Total cholesterol, serum triglycerides, and high-density lipoprotein cholesterol (HDL-c) concentrations, measured in mg/dL, were assessed using an enzymatic colorimetric dry chemistry method (Johnsons & Johnsons, Raritan, USA, VITROS 5600). Low-density lipoprotein cholesterol (LDL-c) was determined using Friedewald’s formula (^23^). Very-low-density lipoprotein cholesterol (VLDL-c), non-HDL cholesterol, the atherogenic index, and the total cholesterol/HDL-c and LDL-c/HDL-c ratios were calculated using designated mathematical formulas. Procedures for collecting dietary intake data were detailed in the study protocol (^14^). Diet adherence was evaluated with the BALANCE dietary index (DI), which assigns points based on adherence to BALANCE food groups, with scores ranging from 0 to 40; higher scores denoted greater adherence (^24^).

The sample size was determined based on convenience, targeting 260 individuals (130 per group) to attain an 80% power for this exploratory analysis. Following the guidelines set forth by Cohen for effect sizes, a medium effect size (d = 0.35) was anticipated for Apo concentrations (^25^). This calculation was performed at a 5% significance level for a two-tailed test. The Shapiro-Wilk test was utilized to verify the normality of the data. Descriptive statistics presented categorical variables through frequencies and continuous variables in terms of means (standard deviations) or medians (interquartile ranges). Treatment effects on continuous outcomes (BALANCE DI components and lipid profiles) were estimated using mixed models with fixed effects for group, time, their interaction, and random intercepts. Between-group comparisons for Apos and ApoB/ApoA-I ratio employed Wilcoxon tests, with paired Wilcoxon tests for within-group comparisons. This approach was justified by the evaluation of the mixed model assumptions, specifically the normality of residuals, which did not hold for Apos and ApoB/ApoA-I ratio, necessitating a non-parametric approach.

For adherence levels to the BALANCE DI, the Chi-square test for linear trend was utilized to compare the groups, considering the ordered nature of the categories. For baseline variables, the Student’s t-test was applied to analyze continuous variables (e.g., mean age, body mass index, and waist circumference), while Pearson’s Chi-square test addressed categorical variables. Statistical significance was defined at *p* < 0.05, with analyses conducted using R software (R Core Team, 2022).

## RESULTS

**Figure 1:**
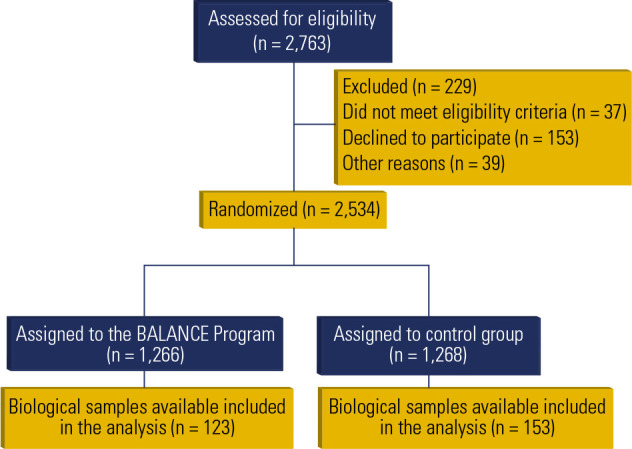
Flowchart of the study based on BALANCE Program data. Adapted from the literature (^14^).

The general characteristics of the study participants are listed in **Table 1.** Notably, the groups displayed similar characteristics at baseline, with no significant differences observed between them. The study sample comprised predominantly men, with a mean age of 63.4 years (SD 8.2). The majority of participants had less than eight years of formal education and a monthly income below USD 939.99. Most were former smokers (54.7%), exhibited a sedentary lifestyle, and had an elevated body mass index. Additionally, a significant proportion of participants were taking medications such as statins (90.6%), anticoagulants (92.4%), and antihypertensive drugs (94.9%). A flowchart of this exploratory analysis is presented in **Figure 1.**

**Table 1:** Baseline characteristics of the participants

Variables	Control (n = 153)	BALANCE (n = 123)	Total (n = 276)	p
Men n (%)	85 (55.6)	78 (63.4)	163 (59.1)	0.23^#^
Age, years (mean ± SD)	63.7 ± 8.7	63.1 ± 7.5	63.4 ± 8.2	0.61^*^
Years of schooling - n (%)				
<8 years	83 (58.9)	69 (57)	152 (58)	0.22^#^
8-11 years	44 (31.2)	46 (^38^)	90 (34.4)	
>11 years	14 (9.9)	6 (^5^)	20 (7.6)	
Household income (USD/month), n (%)				
<543.99	15/142 (10.6)	8/121 (6.6)	23/263 (8.7)	0.54^#^
939.99-544.00	73/142 (51.4)	65/121 (53.7)	138/263 (52.5)	
>940.00	54/142 (^38^)	48/121 (39.7)	102/263 (38.8)	
Smoking status, n (%)				
Non-smoker	60 (39.2)	49 (39.8)	109 (39.5)	0.54^#^
Former smoker	82 (53.6)	69 (56.1)	151 (54.7)	
Current smoker	11 (7.2)	5 (4.1)	16 (5.8)	
Sedentarism, n (%)	94 (61.4)	74 (60.2)	168 (60.9)	0.93^#^
BMI, kg/m^2^ (mean ± SD)	28.8 ± 4.4	29.4 ± 5.3	29.1 ± 4.9	0.32^*^
Nutritional Status, n (%)				
Normal weight	30 (19.6)	22 (17.9)	52 (18.8)	0.85^#^
Overweight	67 (43.8)	52 (42.3)	119 (43.1)	
Obesity	56 (36.6)	49 (39.8)	105 (^38^)	
Waist circumference, cm (mean ± SD)	99.5 ± 11.4	100 ± 12.2	99.7 ± 11.7	0.75^*^
Hypertension, n (%)	144 (94.1)	116 (94.3)	260 (94.2)	0.99^#^
Type-2 diabetes mellitus, n (%)	81 (52.9)	63 (51.2)	144 (52.2)	0.87^#^
Dyslipidaemia, n (%)	135 (88.2)	111 (90.2)	246 (89.1)	0.74^#^
Family history of coronary disease, n (%)	104 (68.0)	93 (75.6)	197 (71.4)	0.21^#^
Previous CVD				
Atherosclerotic stenosis, n (%)	146 (95.4)	118 (95.9)	264 (95.7)	0.99^#^
Stroke, n (%)	10 (6.5)	10 (8.1)	20 (7.2)	0.78^#^
Antihypertensive, n (%)	146 (95.4)	116 (94.3)	262 (94.9)	0.89^#^
Statin, n (%)	137 (89.5)	113 (91.9)	250 (90.6)	0.65^#^
Hypoglycaemic agents, n (%)	66 (43.1)	54 (43.9)	120 (43.5)	0.97^#^
Insulin, n (%)	23 (15.0)	21 (17.1)	44 (15.9)	0.77^#^
Anticoagulant/ antiplatelet, n (%)	140 (91.5)	115 (93.5)	255 (92.4)	0.70^#^

*p*-value:

* Student’s t test;

# Pearson Chi-square test; BMI: body mass index; CVD: cardiovascular disease.

Dietary adherence, assessed using the BALANCE DI, is detailed in **Table 2.** After three years, the intervention group demonstrated higher overall adherence than the control group (mean difference BALANCE-control [95% CI]: 2.09 points [-0.19; 4.37]; *p* = 0.07), attributed to increased consumption of fruits, vegetables, legumes, and low-fat dairy products (mean difference intervention-control in the green group: 0.88 points [0.12; 1.64]; *p* = 0.024) and reduced intake of animal protein and saturated fatty acids, which are prominent in the blue group (mean difference intervention-control in the blue group [95% CI]: 1.3 points [0.27; 2.33]; *p* = 0.027). No significant differences were observed in the other components of the BALANCE DI (yellow and red groups). There was a significant difference in the overall distribution of the BALANCE DI adherence categories between the intervention and control groups at follow-up (*p* = 0.009).

**Table 2:** Adherence to the individual components and total BALANCE dietary index in the study groups

Variables	Control	BALANCE	Main difference (95% CI)^*^ BALANCE-control	p
BALANCE DI				
Green group				
Baseline	4.67 ± 2.72	4.60 ± 2.61	-0.07 (-0.67;0.53)	0.97
36 months	4.73 ± 2.39	5.53 ± 2.42	0.81 (0.18;1.43)	**0.022**
36 m - Baseline (95% CI)	0.05 (-0.45;0.56)	0.93 (0.36;1.5)	0.88 (0.12;1.64)	**0.024**
Yellow group				
Baseline	3.14 ± 3.34	3.48 ± 3.35	0.34 (-0.43;1.12)	0.62
36 months	3.87 ± 3.22	3.99 ± 3.18	0.09 (-0.71;0.9)	0.97
36 m - Baseline (95% CI)	0.75 (0.05;1.45)	0.5 (-0.28;1.28)	-0.25 (-1.3;0.8)	0.64
Blue group				
Baseline	5.34 ± 4.32	5.57 ± 4.25	0.23 (-0.77;1.23)	0.88
36 months	5.52 ± 4.30	6.84 ± 3.91	1.3 (0.27;2.33)	**0.027**
36 m - Baseline (95% CI)	0.2 (-0.64;1.04)	1.27 (0.33;2.21)	1.07 (-0.19;2.33)	0.99
Red group				
Baseline	5.83 ± 3.91	5.71 ± 3.63	-0.12 (-1.01;0.77)	0.96
36 months	5.12 ± 3.63	5.40 ± 3.84	0.28 (-0.64;1.2)	0.8
36 m - Baseline (95% CI)	-0.71 (-1.55;0.12)	-0.31 (-1.25;0.62)	0.4 (-0.86;1.66)	0.53
Total points				
Baseline	18.99 ± 7.57	19.37 ± 7.71	0.38 (-1.35;2.12)	0.89
36 months	19.23 ± 6.59	21.76 ± 7.36	2.47 (0.68;4.26)	**0.013**
36 m - Baseline (95% CI)	0.29 (1.22;1.81)	2.38 (0.68;4.08)	2.09 (-0.19;4.37)	0.07
**Degree of BALANCE DI adherence**				
Baseline				0.44^¶^
Low adherence (0-13 points)	29/153 (19%)	27/122 (22%)		
Moderate adherence (14-26 points)	98/153 (64%)	69/122 (57%)		
High adherence (27-40 points)	26/153 (17%)	26/122 (21%)		
36 months				0.009^¶^
Low adherence (0-13 points)	24/143 (17%)	15/114 (13%)		
Moderate adherence (14-26 points)	97/143 (68%)	63/114 (55%)		
High adherence (27-40 points)	22/143 (16%)	36/114 (32%)		

Values are presented as means ± standard deviation (SD) or absolute numbers (proportions).

* 36m - Baseline (95% CI), mean differences between groups, 95% CI, and *p*-values were estimated using the mixed model.

¶ Pearson’s Chi-square test. The green group is represented by fruits, vegetables, legumes, and low-fat dairy. Yellow group is represented by grains, rice, bread, homemade cookies, vegetable oils, and honey. Blue group is represented by meat, eggs, fish, chicken, homemade cakes and sweets, and butter. Red group is represented by ultra-processed food.

**Figure 2:**
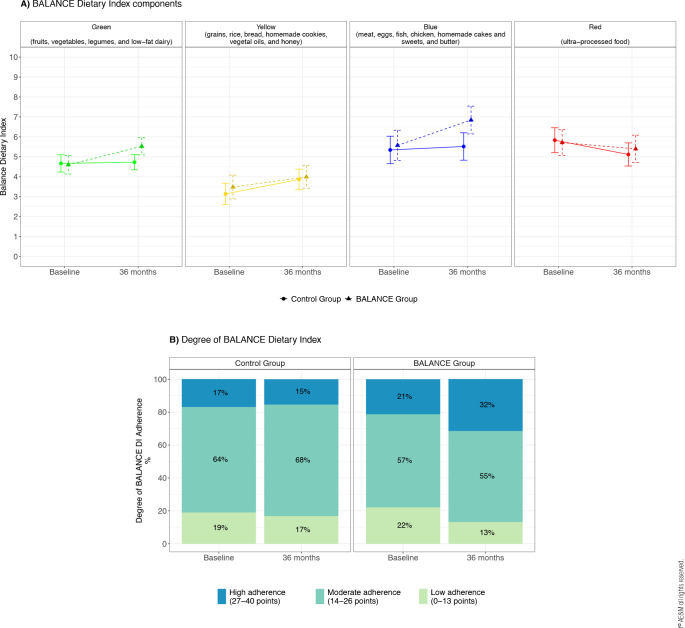
Adherence to the BALANCE dietary index at baseline and after 3 years according to its components **(A)** and degrees **(B)**.

Specifically, participants in the intervention group were more likely to achieve high adherence to the BALANCE DI than the control, whereas participants in the control were more likely to be classified into the low or moderate adherence categories. **Figure 2** illustrates the adherence to the BALANCE DI at baseline and after 3 years, according to its components **(Figure 2A)** and degrees **(Figure 2B)**.

Regarding lipid profiles **(Table 3)**, the intervention group experienced significant reductions in total cholesterol and non-HDL cholesterol concentrations (mean difference BALANCE-control [95% CI]: -9.95 mg/dL [-18.5; -1.39]; *p* = 0.023; -8.86 mg/dL [-17.53; -0.2]; *p* = 0.045, respectively). No significant changes were observed in Apo concentrations **(Table 4)**. Medication usage remained constant throughout the study period (data not shown).

**Table 3:** Lipid profile features at baseline, 36 months, and changes after interventions

Variables	Control	BALANCE	Main difference (95% CI)^*^ BALANCE-control	*p*
Total Cholesterol, mg/dL				
Baseline	160.9 ± 38.3	166 ± 41.7	5.1 (-4.48; 14.68)	0.45
36 months	163 ± 40.7	156.1 ± 41.1	-4.85 (-15; 5.3)	0.52
36 m - Baseline (95% CI)	2.24 (-3.49; 7.97)	-7.71 (-14.06; -1.35)	-9.95 (-18.5; -1.39)	**0.023**
LDL-c, mg/dL				
Baseline	88 ± 31.6	89.1 ± 35.3	1.25 (-7.11; 9.61)	0.94
36 months	89.3 ± 35.1	86.7 ± 40.4	-1.75 (-10.81; 7.32)	0.90
36m - Baseline (95% CI)	1.89 (-3.78; 7.56)	-1.1 (-7.37; 5.16)	-3 (-11.45; 5.46)	0.49
HDL-c, mg/dL				
Baseline	45.4 ± 12.8	46 ± 16.1	0.67 (-2.57; 3.91)	0.85
36 months	45.2 ± 12.0	43.1 ± 11.3	-0.77 (-4.13; 2.6)	0.82
36 m - Baseline (95% CI)	0.12 (-1.28; 1.52)	-1.32 (-2.86; 0.22)	-1.44 (-3.52; 0.64)	0.18
Triglycerides, mg/dL				
Baseline	143.6 ± 107.6	160.2 ± 94.2	16.58 (-5.8; 38.96)	0.26
36 months	138 ± 89.9	140.7 ± 80.2	2.2 (-22.3; 26.7)	0.98
36 m - Baseline (95% CI)	-6.43 (-23.98; 11.12)	-20.81 (-40.2; -1.42)	-14.38 (-40.53; 11.77)	0.28
VLDL-c, mg/dL				
Baseline	28.7 ± 21.5	32 ± 18.8	3.32 (-1.16; 7.79)	0.26
36 months	27.6 ± 18	28.1 ± 16	0.44 (-4.46; 5.34)	0.98
36 m - Baseline (95% CI)	-1.29 (-4.8; 2.22)	-4.16 (-8.04; -0.28)	-2.88 (-8.11; 2.35)	0.28
Non-HDL Cholesterol, mg/dL				
Baseline	115.5 ± 36.4	120.1 ± 38.3	4.67 (-4.35; 13.7)	0.48
36 months	116.9 ± 39.3	112.2 ± 38.9	-4.19 (-13.92; 5.54)	0.60
36 m - Baseline (95% CI)	1.81 (-4; 7.62)	-7.05 (-13.48; -0.63)	-8.86 (-17.53; -0.2)	**0.045**
Atherogenic Index				
Baseline	3.7 ± 1.2	3.8 ± 1.1	0.1 (-0.17; 0.38)	0.66
36 months	3.8 ± 1.2	3.8 ± 1.1	-0.01 (-0.31; 0.28)	0.99
36 m - Baseline (95% CI)	0.02 (-0.15; 0.18)	-0.1 (-0.28; 0.08)	-0.12 (-0.36; 0.13)	0.36
Total cholesterol/LDL-c ratio				
Baseline	1.94 ± 0.44	1.99 ± 0.46	0.05 (-0.05; 0.16)	0.52
36 months	1.93 ± 0.41	1.96 ± 0.55	0.02 (-0.1; 0.14)	0.90
36 m - Baseline (95% CI)	-0.02 (-0.1; 0.06)	-0.05 (-0.14; 0.04)	-0.03 (-0.15; 0.09)	0.62
HDL-c/LDL-c ratio				
Baseline	0.58 ± 0.27	0.59 ± 0.3	0.01 (-0.058; 0.069)	0.98
36 months	0.57 ± 0.26	0.57 ± 0.25	0 (-0.066; 0.071)	0.99
36 m - Baseline (95% CI)	-0.01 (-0.051; 0.032)	-0.01 (-0.058; 0.033)	-0.003 (-0.064; 0.059)	0.93

Values are presented as means ± standard deviation (SD).

* 36m - Baseline (95% confidence interval [CI]); mean differences between groups; 95% CI and *p-*values were estimated using the mixed model. LDL-c: low-density lipoprotein cholesterol; HDL-c: high-density lipoprotein cholesterol; VLDL-c: very low-density lipoprotein cholesterol.

**Table 4:** Apolipoprotein features at baseline, 36 months, and changes after interventions

Variables	Control (n = 153)	BALANCE (n = 123)	Difference (BALANCE-control)^*^			p^¶^
Apolipoprotein A-I, mg/dL						
Baseline	90.2 [61.4-131.5]	88.3 [59.4-140]		-1.40 [-12.93; 10.46]	0.82	
36 months	69.7 [52.6-90.8]	62.5 [47.2-81.6]		-5.32 [-12.09; 1.28]	0.11	
36 m - Baseline	-24.80 [-38.44; -13.34]	-30.28 [-47.81; -17.45]		-4.45 [-20.84; 12.35]	0.59	
Apolipoprotein A-II, mg/dL						
Baseline	56.9 [45.3-79.7]	56.6 [44.5-78]		-1.36 [-6.51; 3.85]	0.61	
36 months	52.2 [43.7-62.8]	53.5 [46.1-65.8]		1.64 [-1.88; 5.38]	0.37	
36 m - Baseline	-5.95 [-9.75; -2.20]	-3.72 [-8.51; 0.60]		1.79 [-3.72; 7.43]	0.56	
Apolipoprotein B, mg/dL						
Baseline	160.5 [111.8-204]	152.7 [118.6-197.7]		-0.57 [-16.35; 14.49]	0.95	
36 months	157.8 [110.2-208]	157.5 [122.2-207.7]		4.00 [-12.79; 20.78]	0.62	
36 m - Baseline	4.37 [-8.97; 17.18]	5.95 [-9.10; 21.12]		2.76 [-17.31; 21.39]	0.78	
Apolipoprotein C-II, mg/dL						
Baseline	14.7 [10.6-21]	15 [10.4-21.6]		0.30 [-1.47; 2.13]	0.74	
36 months	13.7 [10-20.5]	14.5 [10.3-22.2]		0.73 [-0.91; 2.46]	0.38	
36 m - Baseline	-0.35 [-1.88; 1.17]	-0.22 [-1.93; 1.35]		0.04 [-2.15; 2.31]	0.98	
Apolipoprotein C-III, mg/dL						
Baseline	37.5 [26.8-52.5]	34.5 [25.2-53.5]		-0.98 [-5.35; 3.38]	0.66	
36 months	36.8 [26.3-49.2]	36 [26.7-53.8]		1.58 [-2.80; 6.15]	0.49	
36 m - Baseline	-0.21 [-3.58; 3.32]	0.23 [-3.79; 4.40]		0.35 [-5.01; 5.60]	0.89	
Apolipoprotein E, mg/dL						
Baseline	2.7 [1.6-4.2]	2.5 [1.6-3.9]		-0.16 [-0.54; 0.21]	0.35	
36 months	3.2 [2.5-4.7]	3.7 [2.7-4.5]		0.16 [-0.22; 0.53]	0.41	
36 m - Baseline	0.54 [0.21; 0.86]	0.84 [0.49; 1.21]		0.30 [-0.18; 0.79]	0.21	
ApoB/ApoA-I ratio						
Baseline	1.7 [1-2.4]	1.6 [1-2.6]		0.02 [-0.25; 0.31]	0.91	
36 months	2.3 [1.5-2.9]	2.4 [1.8-3.2]		0.22 [-0.04; 0.48]	0.10	
36 m - Baseline	0.58 [0.33; 0.81]	0.65 [0.39; 0.93]		0.08 [-0.26; 0.47]	0.64	

The values are presented as medians [interquartile range].

* Intra-group difference (baseline and 36-month comparison) and 95% CI were estimated using the paired Wilcoxon test.

¶ Differences between groups 95% CI and p-values were estimated using the Wilcoxon test. ApoB: apolipoprotein B; ApoA-I: apolipoprotein A-I.

## DISCUSSION

Following three years of follow-up, adherence to a healthier diet, as demonstrated by the BALANCE Program, was notably higher in the intervention group. This increase in adherence was attributed to an enhanced intake of cardioprotective foods from the green group and a decreased intake of foods from the blue group. Nonetheless, these changes in dietary behavior were modest and insufficient to significantly influence clinical lipid profiles and Apo concentrations.

Consistent with large meta-analyses, our findings suggest that adopting healthy dietary patterns alone often falls short of significantly impacting lipid profiles in secondary prevention settings, a conclusion supported by excluding Apo analyses in these prior studies (^26^). The observed effects of the BALANCE Program on total and non-HDL cholesterol levels in this exploratory analysis could partially be ascribed to increased vegetable consumption (^27^) and a reduction in the intake of animal-based foods (^28^). Nonetheless, significant reductions in lipid profile markers likely result from not only a marked increase in the consumption of vegetables, leafy greens, and fruits but also the inclusion of other beneficial foods and nutrients such as nuts, vegetable oils, monounsaturated fats, and phytosterols (^29^,^30^), as well as a reduction in saturated fat-rich foods predominantly found in the blue group, which includes animal-based foods such as red meat (^28^). Despite these factors, we propose that the phenomenon of regression to the mean more aptly explains our findings, especially considering the higher baseline values in the intervention group.

Exploring further, the impact of dietary behavior changes on the study sample - who increased their intake of plant-based foods (green group) and reduced their intake of animal-based foods (blue group) - merits consideration. The guidance of the BALANCE program closely resembles that of a plant-based diet, which has been linked to significant reductions in cholesterol (^31^) and ApoB (^32^). However, the paucity of studies assessing this association in secondary prevention contexts is notable, and although our study observed a reduction in cholesterol among patients in the intervention group, no significant difference in ApoB levels was found.

The DASH and Mediterranean diets are widely recognized as fundamental nutritional guidelines for cardiovascular prevention. Previous research in primary prevention settings illustrated that short-term adherence (3 months) to the Mediterranean diet could decrease non-HDL cholesterol and ApoB levels while increasing ApoA-I levels (^7^). A randomized trial (n = 52) evaluating both Mediterranean and lacto-ovo-vegetarian diets over 3 months revealed positive effects on various Apos, especially among women and those over 50 years old or with fewer than three cardiovascular risk factors (^8^). However, in alignment with our results, a long-term study with the Mediterranean diet among Spaniards with previous CVD did not demonstrate changes in Apo levels (^33^).

Concerning the traditional DASH diet, one study with healthy individuals reported a decrease in ApoA-I levels after 3 weeks, with no alterations in ApoB (^9^). In contrast, a DASH dietary pattern enriched with carbohydrates, proteins, or unsaturated fats positively influenced ApoB and ApoC-III levels after a 6-week intervention in healthy participants (^34^). These varied outcomes across trials may be attributed to differences in population profiles, study sample sizes, follow-up durations, and diet diversity. Moreover, individuals in secondary prevention frequently use multiple medications, such as statins, which are known to affect Apos (^35^). Additionally, our study’s lack of intermediate time point data, such as at 3 or 6 weeks, to assess the intervention’s effects over shorter periods, with our data only covering a 3-year follow-up, further complicates these observations.

ApoA-I and ApoB are deemed more discriminative markers for defining cardiovascular risk due to their lower analytical variability compared to HDL-c and LDL-c, respectively (^5^). Expected values for ApoA-I and ApoB in primary prevention populations typically vary at 90-170 and 56-162 mg/dL for women and 107-214 and 51-171 mg/dL for men, respectively (^36^). Our study found higher baseline ApoA-I concentrations, which is consistent with the secondary prevention setting (^33^), suggesting higher anticipated HDL-c values. Despite this, HDL-c concentrations remained unchanged over 36 months, while ApoA-I levels fell by nearly 65%. Although HDL-c and ApoA-I levels are expected to correlate positively, a U-shaped association between mortality/CVD incidence and ApoA-I levels, independent of HDL-c, is apparent (^37^). Exceptionally high ApoA-I levels may indicate increased disease risk or severity, which is pertinent given the high cardiovascular risk profile of participants in the BALANCE Program.

After three years, no significant difference in the consumption of foods from the yellow and red groups according to the BALANCE DI was noted. These groups are defined by foods high in carbohydrates and trans fatty acids, respectively, which are closely related to specific Apos associated with triglyceride-rich and atherogenic particles (^38^,^39^). Despite an increase in vegetable and fruit intake in the intervention group, this was not sufficient to affect Apos, due to the unaltered intake of macronutrient-rich foods like carbohydrates and fats throughout the study.

Despite our promising findings, this study has limitations. Although the sample size was determined through power calculations, it may still be prone to type II errors. The BALANCE Program was pragmatic and not initially designed to explore the research question posed in this exploratory analysis, nor was it established as a biorepository for future blood sample analyses. The biorepository was established after most participants were recruited, leading to inconsistent collection and storage of blood samples. This inconsistency also explains the varied sample sizes between the intervention and control groups despite their similar characteristics **(Table 1)**. Patients with low Program adherence, as well as those who died during the study, were excluded from this sub-analysis, as it necessitated blood samples from both baseline and the 3-year follow-up. Consequently, the generalizability of our findings may be limited, as they do not represent individuals with lower adherence to the protocol or those with more severe forms of CVD. Nevertheless, the study’s focus on a secondary prevention sample and the extensive follow-up period of three years stand as significant strengths.

In conclusion, after three years of follow-up, the BALANCE Program did not significantly impact plasma Apos concentrations in a secondary cardiovascular prevention context, despite improvements in diet quality and modest shifts in lipid biomarkers. Further research is warranted to examine the effects of different dietary patterns on Apos within the scope of secondary cardiovascular prevention.
